# Clinical Conversations on Diseases of the Skin

**Published:** 1876-11

**Authors:** L. D. Buckley


### Clinical Conversations on Diseases of the Skin.

By L. D. Buckley, M. D. Reported by Robert Campbell, M. D., Clinical Assistant.

Case I. *Psoriasis punctata.* This man, aged 32, exhibits an eruption, which, while it should offer no difficulty in diagnosis, might on superficial observation perplex one not familiar with the disease in all its features. Psoriasis is very rightly associated in the minds of most physicians with circular patches of diseased tissue, reddened bases with an abundance of silvery imbricated scales, readily detached, the eruption being most marked upon the elbows and knees; the patches are usually thought of as of good size, from an inch upward in diameter. The eruption on this man, as you see, has spared the knees entirely, the elbows being very slightly affected, while a large share of the body is thickly sprinkled with these small reddish dots of various sizes, most of them not larger in diameter than that of a split pea, the largest being hardly as large as one's thumb-nail; the smallest hardly larger than a large pin-head. Moreover most of them present very little of scale. But on scraping each of these little spots you get a small amount of the same silvery scale obtainable from large patches, and on further removing this we find at least a little pellicle which slips off, and beneath we have the exposed corium which bleeds with slight irritation.

Now what are the features distinguishing this case to be psoriasis, for this disease may be confounded with the scaly syphiloderm, chronic scaly eczema, tinea circinata, and possibly (though hardly in this country), with lupus erythematosus.

*Syphilis* is excluded by the absence of specific history and by the fact that he has had the eruption on several occasions dating back five years. A syphilitic eruption never repeats itself in exactly the same form. But the appearance of the eruption itself is quite characteristic to the practised eye; you will never get such silvery scales on scraping a syphilitic eruption, nor just this bleeding state of the corium on scraping them off forcibly. The size of the single spots varies greatly from these minute, almost pin-head specks to these others, half of three-quarters of an inch in diameter on the body; those of syphilis are more uniform in size and medium between the extremes, they are more elevated than the flat squamous syphiloderm, less so than the later variety of tubercular character. The color is that of psoriasis and not of syphilis, the difference I cannot express in words truly, (for the historic copper-colored hue of syphilitic eruptions is somewhat of a myth,) you will learn to recognize the psoriasis shade by practice.

One could hardly mistake this eruption for any form of *eczema* if the entire eruption was exposed (as it always should be); scaly eczema almost always has had a previous moist stage, this has always been dry. I have never known eczema to present so many isolated spots and as small. I have seen ringworms, *tinea circinata* covering the entire body, but such cases are very rare and the great extent of this eruption would readily distinguish it from the common forms, as also would a microscopic examination of the scales. I have never seen a case of *erythematous lupus* which could be confounded with psoriasis although authors mention it, nor do I know of any other disease which you could reasonably suspect this to be, unless it were *tinea* or *pityriasis versicolor*. But here the patches, which may be small and circular and extend even down upon the arms, are always brownish-fawn color, without a shade of red, and the microscopic demonstration of the vegetable parasite, *microsporon furfur* is very simple, and certain, when the tinea is present. The man says that this eruption appeared, on the last occasion, after bathing in salt water; I have a number of times known this small and very generally distributed eruption of psoriasis occur as it were in an acute manner after bathing in the sea, or after chilling of the surface, while we know that the chronic variety, with a tendency to large patches, comes on most unaccountably, and seldom can any local or special exciting cause be found. Psoriasis exhibits well marked and unmistakable evidences of a constitutional origin, although it may be difficulty to trace it in every case, especially among the poor, and the sudden appearance of diffuse eruption following a definite cause, as severe sea bathing, but shows the intensity of the constitutional disturbance responding to general stimulation of the integument.

We shall give this man tar internally, combined with an alkali, in the following prescription, which has been of service in other cases; it is the "*liquor picis alkalinus*" which you have often seen prescribed here externally with such success. The formula is as follows: ℞. Picis liquidæ, ℥ ij.; Potass. Causticæ, ℥; Aquæ, ℥ v. M. Dissolve the potossa in the water and add slowly to the tar, with friction in a mortar. Commence with fifteen drops and increase slowly to half a teaspoonful after meals in considerable water.

Case II. *Untreated syphilis, late tubercular ulcerations.* The case of this German, aged 25, is interesting, as showing the course of syphilis when left to itself, such cases as this demonstrating clearly the untenability of anti-mercurialistic doctrines, for, you know, certain writers, happily few and obscure, have urged that the ulcerations observed in the subject of Syphilis are the result of the mercury and not of the disease. This man contracted a chancre in 1868, which was treated locally and disappeared, and he acknowledges no syhilitic history except sore throat, rheumatism and headache, *and he has never received any internal treatment* for these or other syphilitic symptoms.

Two years ago the present eruption began near the inner malleolus of the right leg, and has continued in increasing severity, in spite of various local applications, until now you see the entire left leg, except the upper quarter, is involved in a mass of ulcerating lesions, whose sickening odor at his first visit filled the room. This diseased surface is made up of masses of tubercular deposit, with cicatrical tissues between each of these foul ulcers with sharp livid edges and pultaceous base, representing separate syphilitic neoplasms. On the right side of the head and forehead, you see similar ulcerating mass and a number of cicatrices, entirely devoid of hair.

This man has been under treatment at one of the Homœopathic Public Institutions for some months, where the sores had been subjected to constant local treatment, but no internal remedies whatever have been employed: they have steadily gotten worse, until now he is indeed an object of pity.

The elements of diagnosis in the present case are, the large amount of ulcerative surface, its character and distribution. Seldom will one see such a leg as this apart from syphilis; varicose and eczematous ulcers of the leg are very rarely so extensive as this, their surface is not so riddled by ulceration, the base of the ulcers is more clean, with less of this necrotic tissue; moreover, the ulcerations and cicatrices of the scalp are evidently of the same character, and as it would be impossible for them to be eczema, from their appearance in this locality, those on the leg must also be of another origin. This combined picture, then, can be afforded by no other disease but syphilis; it is hardly possible to see how the true nature of this disease could have escaped recognition so long.

We gave him no local treatment, but allowed him to continue what he is now using, a carbolic ointment, in order to demonstrate how completely such ulcerative processes are under the control of the proper constitutional measures. One week ago he received our ordinary mixed treatment, containing 1-32 grain of bichloride of mercury, abcut eight grains of iodide of potassium, two grains of iron, a little tincture of nux vomica, and compound tincture of cinchona, in each dose, after meals, thrice daily, and those who remember his state then, will see the wonderful power of the treatment. The offensive odor has entirely departed, some of the diseased surfaces have taken on a healthy action. In such cases you can pretty confidently predict a perfect and quite speedy recovery.

Case III. *Warts cured by arsenic internally.* This girl, of 11 years, exhibits now nothing of importance diagnostically, there being but a single ordinary wart on the inside of the third finger of the right hand. The case is interesting, however, because, when she first came here, June 2, she had ten of these deformities, a number being on the face; and these have all disappeared, except this one, solely under the internal use of arsnic. Three weeks after her visit, it is recorded that several of the warts had disappeared.

CASE IV. *Scabies, difficult of recognition* I venture to say that very few, other than those specially practising dermatology, would recognize that this young man, aged 19, has scabies; and yet the evidences are so clear that there cannot be a shadow of a doubt in reference to the true nature of the disease. The unusual features in the case are, that no history can be gotten of contagion (for generally the disease can be traced), and that there is such a very slight development of the eruption on the parts commonly recognized as liable to be affected. If we look on the sides of and between the fingers we find almost no eruption, a few small papules presenting nothing characteristic; except here, between the thumb and forefinger of right hand, we find a distinct and well marked *cuniculus*, or *furrow*, and another not quite so clear. These minute, blackish, irregular, pretty sharply defined, curved lines are the one sure clinical sign of the disease, and the extraction of the acarus from them, and its demonstration beneath the microscope, would furnish evidence beyond doubt, even for the most skeptical. There are some papules and vesicles on the flexor surface of the wrists, but no distinct cuniculi or furrows can be seen here.

You heard me ask him almost immediately after inspecting the hands and the wrists, whether he had any eruptian or any itching about the genitals, and here, upon the penis, we find the same small, scratched papules, and on the glans itself a cuniculus is plainly visible. I think that enough attention has not been called to the fact that in the male the penis or scrotum, or both, will very generally be affected in scabies. The constant touching these organs in answering the calls of nature, and the habit some have of handling the genitals when in bed, give abundant opportunities for the acari to pass thence from the hands, where we must believe they generally find their first lodgement. In the female it is very common to find the breasts thus affected in scabies, and some French writer has claimed that eczema of the breast in women who are neither nursing nor pregnant, is always dependent upon scabies, a rather too broad statement. These few papules, therefore, upon the sides of the fingers, between the right thumb and forefinger, and on the penis, are quite sufficient to characterize the disease of scabies, even if the cuniculi were absent, for I do not know that such a combined picture is furnished by any other disease of the skin. This, you will understand, is a very mild case of scabies; there are very few of the results of scratching, pustules, &c., though he says that the whole body itches much at times: the disease is comparatively recent, he having perceived it only two weeks. It will yield completely, I expect, to a thorough warm bath, with yellow soap, and then a patient friction with the sulphur ointment of the U. S. Pharmacopœia for a few nights, leaving the ointment on. This fellow's skin is pretty hard, and there are no excoriations, or such a procedure would be much too harsh; for scabies in women and children, you will see me prescribe storax, two drahms to the ounce, with perhaps, a little sulphur, say a drahm or so.

CASE V. *Venous dilation in the skin.* It is rather difficult to locate the exact position in dermatological nosology, of the lesion on this woman's leg. It is evidently the same process as occurs in the ordinary varicose veins, indeed this latter condition is also found to a very marked degree on her legs; but here on the left, extending over a distance of about eight inches up from the internal malleolus, by three or four wide at its broadest part, we find an almost continuous varicosity of very small veins or capillaries, and these are seen to be very near the surface, and light pressure over the dark purplish-brown surface (which causes much pain) shows that moving blood is contained beneath a very thin covering. On the upper portion where the disease has seemed to be spreading, a sort of purpuric state has preceded this dilation, and we have up here simply a staining, as though a diapedesis had occurred, or a minute capillary rupture, for this coloration is not altered at all by pressure.

What I want to call particular attention to is the great comfort and benefit she has derived from painting the leg once or twice daily, two or three coats, with flexible collodion (Collodii ℥ i.; Olei Ricini, gtt. v. M.). The tenderness has now very largely disappeared and she can stand on the leg with ease; the tendency to increase seems also to be somewhat checked. She has been taking half a drachm of the fluid extract of ergot, three times a day, for some little time, but I have some doubt as to the amount of good effected thereby in this disease. Certain it is, however, that she has obtained great assistance from the use of the collodion, as suggested by my friend Dr. Engelsted of Copenhagen, when at the clinic a week ago.—*Archives of Dermatology*, *Oct.*, 1876.

